# Fracture of Cervical Vertebra

**Published:** 1836

**Authors:** Hiram A. Prout

**Affiliations:** Tuscumbia, Alabama


### Art. II.—Fracture of a Cervical Vertebra.

*Case of Fracture of the Fifth Cervical Vertebra, without displacement of the body of the bone*, by Hiram A. Prout, M. D., of Tuscumbia, Alabama.

Abraham, a man of color æt. 30 — while wrestling with a companion, was thrown suddenly upon his neck, by having his feet tripped from the ground. The fall was immediately succeeded by a loss of motion and feeling in the shoulders and arms, in the walls of the chest and abdomen, and in the lower extremities. Though there was an entire loss of sensibility to the impression of external agents, he was subject to occasional and severe pains in all the paralyzed parts, and to constant and lancinating pains in his arms and shoulders. There was no external mark of injury over the spinous process of the fifth cervical vertebra; but a distinct crepitus was perceptible on pressure. His breathing was short and extremely laborious, being carried on alone by the action of the diaphragm. The muscles of the head and neck, above the origin of the phrenic nerve, maintained their integrity. His pulse and the temperature of his body were unaffected, unti near the close of life; which occurred within 48 hours after the accident. On the day subsequent to the injury, he was affected with retention of urine and great abdominal distention; notwithstanding the peristallic action of the bowels had been excited and met with no resistance from the paralyzed sphincter. There was no distention of the corpora cavernosa.

Dissection.—The rim or arch of the fifth cervical vertebra was fractured in three places, and the spinous process, with a part of the arch, was driven in upon the spinal marrow. There was a slight effusion of blood, between the sheath of the spinal marrow and the bone, and a considerable effusion between it and the substance of the cord. There was no material lesion of the medulla or of its investing membranes; and the body of the bone was not fractured or displaced, at the intervertebral junction.

Observations.—In this case, the diagnosis was readily established, by the accompanying symptoms and a reference to the physiological relations of the parts. The absence of the regular elevation and depression of the ribs, which attend normal respiration; the increased action of the diaphragm; and the want of contraction in the abdominal muscles, all referred the seat of injury, to some point below the origin of the fourth pair of cervical nerves; whilst the entire loss of motion and sensibility to external impressions, in the arms and shoulders, clearly indicated that the fifth vertebra of the neck was the seat of fracture or displacement, or perhaps of both; as one seldom occurs without being accompanied by the other. This latter diagnostic distinction is based on an observation offered by Sir Astley Cooper, when speaking of accidental lesions of the spine, where he remarks, "if a fracture occurs at the sixth or seventh cervical vertebra, the patient has some feeling and powers of motion" in the arms; "but if at the fith little or none." It seems that the same distinction had been noticed by Galen, who, in treating of palsies from other causes, observes that when the origins of the sixth and seventh pairs of nerves are involved in disease, some sensibility and voluntary motion are still manifest in the shoulders and arms.

It is unnecessary to say, that the truth of these positions and the diagnosis based upon them were amply confirmed by the appearances on dissection.

The constant and intense pain, in the muscles supplied by the palsied nerves, which resulted no doubt from a mere perversion of function, is a symptom of fracture or displacement of the vertebra, which Sir Astley Cooper seems to have overlooked, in his very excellent and accurate account of the phenomena accompanying injuries of the spine. That this symptom is one of not unfrequent occurrence, in palsies originating from spinal lesions, may, we think, be infered from the assertions of writers on palsies in general.

Dr. Cooke, in his work on Nervous Diseases, observes, that pain in the muscles is not an uncommon symptom of palsy, particularly of the shoulders and arms.

Dr. Abercrombie, in his observations on inflammation of the hemispheres of the brain, remarks that in palsies connected with these inflammatory affections, there is sometimes, especially in the early stages, violent pains in the affected limbs. In the case under review, the pain was evidently not the result of inflammatory action, as it immediately succeeded the accident, and was consequently manifested before inflammatory action could establish itself in the injured part.

The observations of these authors satisfactorily confirm the fact that palsies, resulting from diseased action in the brain and spinal marrow, are sometimes accompanied by severe pains in the paralyzed limbs: while the cases, recorded in the *Lecons Oracles* of M. Dupuytren, showing that rheumatic pains and stiffness of the neck have been mistaken for luxations of the vertebra, render it certain, that the same symptom, occasionally at least, attends palsy from accidental injury of the spinal marrow. We do not insist on this symptom as being important to the accuracy of diagnosis in these lesions, but as interesting only, so far, as it may contribute to a knowledge of the phenomena which are manifested in traumatic palsy; and of the physiological relations of the body in general.

In the case recorded by Lallemand, as quoted by Dr. Abercrombie, the pain was confined to the nerves of touch: in the case under consideration there was no morbid external sensibility; the pain was more deeply seated, and seemed to affect the nerves, which supply the muscles of animal life. The cause of this difference, we shall not attempt to define, as we cannot expect to arrive at certain conclusions, with our present imperfect knowledge of the intimate relations of the nervous system.

In the case before us, the distinction, so correctly established by Bichat, between the nervous functions of animal and organic life, was beautifully illustrated. The heart, the arteries and the veins, the stomach and the bowels, all remained unimpaired in any appreciable degree, until an extension of the lesion of the spinal marrow destroyed the function of the phrenic nerves, and consequently, completely suspended the imperfect respiration; now maintained alone by the action of the diaphragm. Had the phrenic nerves remained unaffected, it is obvious, that the patient might have survived several days longer. The failure of the vital functions did not proceed, so much from a want of organic action in the different parts of the body, as from an entire loss of function in the system of animal life.

The want of contraction the coats of the bladder and the relaxation of the sphincter ani manifested, still farther, the correctness of the distinction between the organic and animal functions of the body.

We could dilate on this subject, but deem it more proper to submit the case and these observations to the consideration of others, who may be more immediately engaged in tracing out the physiological relations of the nervous system.

*Tuscumbia, Alabama, August 29th*, 1836.

